# Percutaneous aspiration embolectomy of superior mesenteric artery using a 5MAX ACE reperfusion catheter

**DOI:** 10.1259/bjrcr.20160039

**Published:** 2016-11-02

**Authors:** Hironori Kikuchi, Ichiro Ikushima, Hajime Ohta, Shunrou Uchinokura, Gou Irisa, Toshinori Hirai, Yasuyuki Yamashita

**Affiliations:** ^1^Department of Radiology, Miyakonojo Medical Association Hospital, Miyakonojo, Japan; ^2^Department of Neurosurgery, Miyakonojo Medical Association Hospital, Miyakonojo, Japan; ^3^Department of Radiology, Miyazaki University School of Medicine, Miyazaki, Japan; ^4^Department of Radiology, Kumamoto University School of Medicine, Kumamoto, Japan

## Abstract

Acute mesenteric ischaemia is a rare abdominal emergency that commonly results in bowel infarction and has a very high mortality rate. Therefore, prompt recognition and treatment are crucial for a successful outcome. A thrombectomy for embolism in the mid portion of the main trunk of the superior mesenteric artery (SMA) is proposed. A near-complete thrombi removal from the main trunk of the SMA was achieved by using a 5MAX ACE reperfusion catheter, which was designed for treating cerebral embolism. This is the first report describing the treatment of acute mesenteric ischaemia using this catheter. Percutaneous aspiration embolectomy with this catheter is a useful modality for recanalization of embolic occlusion of not only the cerebral artery but also the SMA.

## Introduction

Acute mesenteric ischaemia (AMI) is a serious but relatively infrequent abdominal emergency that is characterized by sudden interruption of intestinal blood flow that commonly results in bowel infarction and an increased risk of cardiovascular events, particularly in the elderly. The most common cause of AMI is embolism (40–50% of cases).^1^ The majority of mesenteric emboli originate in the heart, most commonly in patients with atrial fibrillation. Despite advances in accurately diagnosing and treating AMI over the past decades, prognosis remains poor, with an in-hospital mortality rate of 59–93%.^2^ Early recognition and treatment are crucial for successful outcome. Because delayed diagnosis results in intestinal infarction and gangrene that cannot be reversed by restoration of blood flow, the treatment of choice has been surgical laparotomy with thrombectomy.

Alternatively, the use of percutaneous thrombectomy has been reported.^3^ In several endovascular strategies, such as percutaneous aspiration embolectomy, thrombolysis, percutaneous transluminal angioplasty, primary superior mesenteric artery (SMA) stenting, the main percutaneous method is aspiration embolectomy, in which the thrombus is removed by suction.

The use of newer thrombectomy devices in patients with severe acute ischaemic stroke can achieve better recanalization rates in much shorter times than with the use of older devices. The 5MAX ACE reperfusion catheter (Penumbra, Inc., Alameda, CA), a new large bore aspiration catheter that is available for vessel recanalization, was launched in July 2013.^4^ We report the use of this catheter for successfully treating a patient who had developed AMI.

## Case report

**Figure 1.**
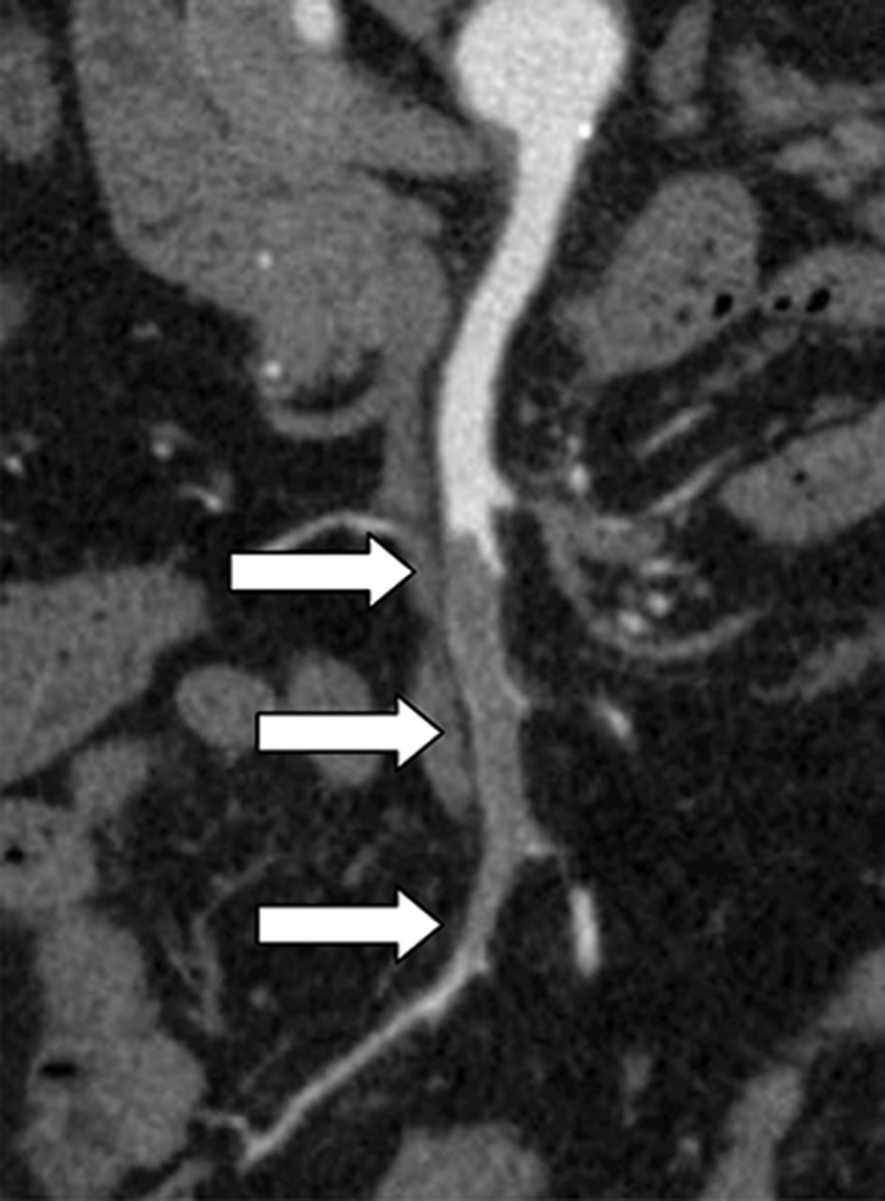
Curved multiplanar reconstruction of CT angiography showing a segmental occlusive acute embolism of the mid portion of the main trunk of superior mesenteric artery proximal to the ileocolic artery (arrows).

A 72-year-old male was admitted to a tertiary academic hospital because of sudden severe central abdominal pain. Except for hyperlipidaemia, no abnormal laboratory findings such as lactate, white blood cells, amylase, lipase and liver enzymes were present. His vital signs were normal. His medical history included atrial fibrillation and Type 2 diabetes mellitus. He had not taken warfarin for 2 weeks. Contrast-enhanced CT angiography (CTA) revealed segmental occlusion of the mid portion of the main trunk of the SMA proximal to the ileocolic artery (Figure 1). Proximally and distally from the occlusion SMA was of normal size, suggesting an embolism rather than a thrombosis. No signs of irreversible bowel wall ischaemia such as bowel wall thickening or pneumatosis were evident. It was considered that thrombolysis could not be effective because of the largesize of the embolus. Because the embolus was located in the main trunk of the SMA, it was expected that aspiration embolectomy would be effective.

After careful consideration, we obtained consent from the patient under an institutional review board-approved protocol for using a 5MAX ACE reperfusion catheter for endovascular revascularization. A decision for endovascular treatment *via* the transfemoral approach under local anaesthesia was reached in concordance with the surgeons.

**Figure 2.**
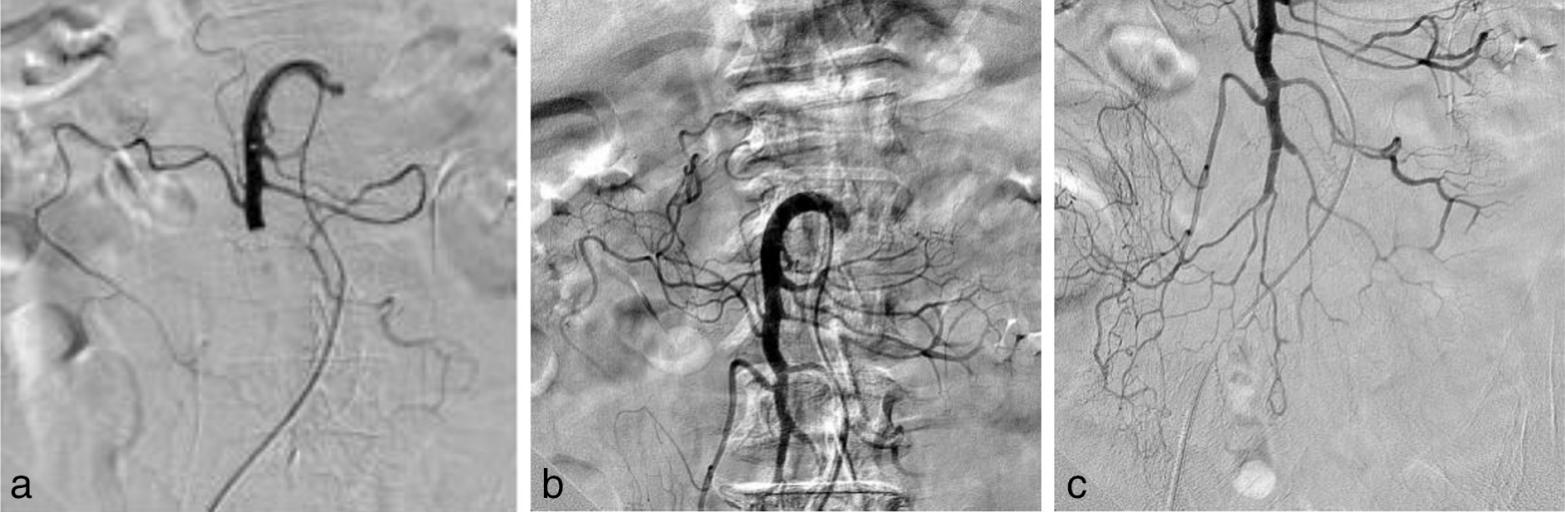
Successful treatment of a segmental complete thrombotic occlusion of the mid portion of the superior mesenteric artery trunk by primary aspiration thrombectomy. (a) Superior mesenteric artery arteriography showing a complete thrombotic occlusion of the mid portion of the main trunk. (b, c) Complete removal of the thromboembolism from the main trunk. There was no migration of thrombi after repetitive primary aspiration thrombectomy using the 5MAX ACE reperfusion catheter.

**Figure 3.**
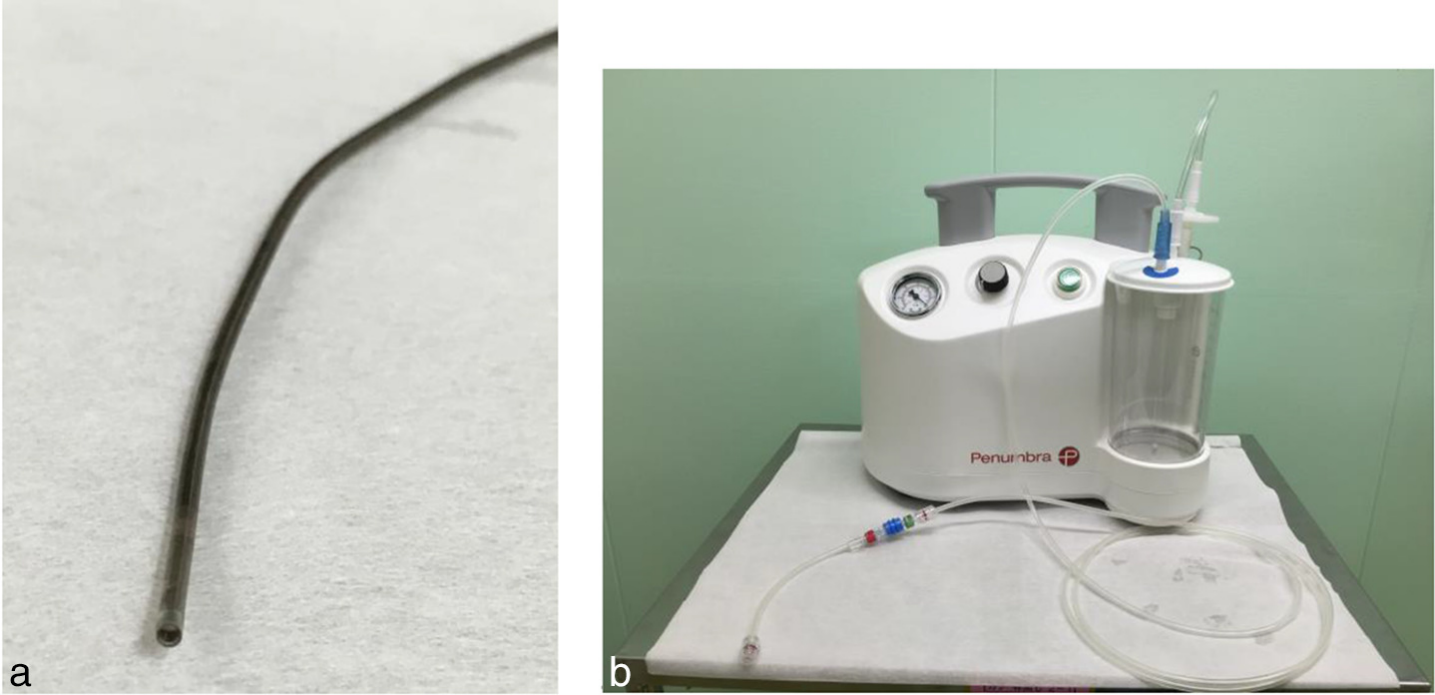
Penumbra system. (a) 5MAX ACE reperfusion catheter, which was designed for treating cerebral embolism. (b) Penumbra aspiration pump and aspiration tubing.

Access was established *via* the right common femoral artery and a 6 French introducer sheath system (Terumo Corporation, NJ). Selective catheterization of the SMA was performed using an angled glide catheter (4 French; Cordis Corporation, Miami Lakes, FL) and a 0.035-inch long guidewire (Radifocus Guidewire M; Terumo Corporation, Tokyo, Japan). Initial SMA angiography confirmed acute occlusion in the mid portion of the SMA trunk (Figure 2a). After infusing 4000 IU of heparin, the 5MAX ACE reperfusion catheter exchange was performed and it was advanced to the face of the clot over the 0.035-inch guidewire. Once the 5MAX ACE was immediately adjacent to the clot, the guidewire was removed. Mechanical suction was applied to the 5MAX ACE using a Penumbra aspiration pump and aspiration tubing (Penumbra, Inc., Alameda, CA) (Figure 3). Once a good seal was obtained, the 5MAX ACE was slowly withdrawn, while maintaining aspiration. Afterwards, the 5MAX ACE was removed from the body, and the guide sheath was opened to enable back bleeding of possible clots. The catheter was then flushed forward once it was confirmed that there were no more clots. Final contrast injection after five passes demonstrated near complete resolution of the thrombus from the main trunk of the SMA (Figure 2b,c). No adjunctive endovascular procedure was applied. The patient’s symptoms almost immediately improved after the procedure, and he no longer complained of postprandial abdominal pain. He remained admitted after the procedure for 3 days and was instructed to continue taking dabigatran etexilate after discharge. CTA on the day after the procedure revealed that the thrombus of the SMA had resolved. A 3-month follow-up excluded any clinical symptoms of abdominal ischaemia.

## Discussion

AMI is a life-threatening condition that commonly results in bowel infarction. The reasons for the high mortality rate are late detection because of non-specific clinical findings, infrequency of the condition and complex surgical treatment. Moreover, diagnosis of AMI is dependent on the ability of the attending physician to suspect and recognize AMI. In patients with acute abdominal pain and a disproportionate lack of clinical, laboratory and ultrasound findings, AMI should be considered, particularly with the presence of risk factors, such as atrial fibrillation, general atherosclerosis or hypercoagulability. Once AMI is suspected, CTA should be performed immediately to proceed with a suitable therapy as soon as possible. If there are clinical or CT signs of bowel necrosis, urgent surgery should be performed. However, if there is no clear evidence of bowel necrosis, endovascular treatment presents a promising alternative.

To the best of our knowledge, there are relatively few reports of mechanical thrombectomy of an SMA embolism. Moreover, this is the first case report of treatment of an SMA embolism with a 5MAX ACE reperfusion catheter. Higher quality recanalization, shorter procedure time and improved safety can be achieved in patients with AMI using the 5MAX ACE than with catheters of similar size.^8^ The 5MAX ACE has an inner lumen diameter of 0.068 inches and an outside diameter of 6.0 French proximally, and a tapered distal lumen with an inner diameter of 0.060 inches and an outside diameter of 5.4 French. Given the large inner lumen, large pieces of a clot can be extracted. Despite its size, the 5MAX ACE was easy to navigate because of 12 transition zones that enable outstanding force transmission and exceptional kink resistance. Moreover, the advanced polymer provides flexibility for superior tracking and reduces the risk for vessel trauma. Furthermore, nitinol round wire reinforcement maintains lumen integrity.

Direct aspiration involves the placement of a large bore aspiration catheter on the face of the clot and aspirating until the device becomes occlusive. This technique has become increasingly feasible and is currently considered the primary method for mechanical thrombectomy.^9^ The main advantages of this technique are rapid and effective removal of large thrombi without the requirement for local thrombolysis and its minimal invasiveness, thus avoiding the complications associated with surgery and lower potential risk of distal embolic event.

Experiences with the use of several other devices to remove blood clots in the SMA are reported, including the Merci retrieval device, which was also designed for treating cerebral embolism.^10^ However, the Merci retrieval device was found to be inferior to the 5MAX ACE reperfusion catheter based on a modified Rankin scale score of ≤ 2 at 90 days; revascularization to thrombolysis in myocardial infarction score 2–3; and mortality at 90 days by treatment of the cerebral embolism.^11–12^ Therefore, the Merci retrieval device has not been used in the treatment of cerebral embolism recently.

In conclusion, a successful recanalization of an acute thrombotic occlusion of the SMA was achieved in this case. Percutaneous aspiration embolectomy with the 5MAX ACE reperfusion catheter is a useful modality for recanalization of an embolic occlusion of not only the cerebral artery but also the SMA and appears to be a safe and effective alternative to open surgery in certain cases.

## Learning points

1. AMI is a life-threatening condition that commonly leads to bowel infarction. Prompt recognition and treatment are crucial for a successful outcome.
2. 5MAX ACE reperfusion catheter was demonstrated to be a viable alternative procedure for emergent SMA revascularization. Higher quality recanalization, shorter procedure time and improved safety are expected by use of this catheter.

## Consent

Written informed consent for the case to be published (including images, case history and data) was obtained from the patient for publication of this case report.

## References

[1] Shih MC, Hagspiel KD, Hagspiel KD CTA and MRA in mesenteric ischemia: part 1, role in diagnosis and differential diagnosis. AJR Am J Roentgenol 2007; 188: 452–61. 17242255 10.2214/AJR.05.1167

[2] Schoots IG, Koffeman GI, Legemate DA, Levi M, van Gulik TM Systematic review of survival after acute mesenteric ischaemia according to disease aetiology. Br J Surg 2004; 91: 17–21. 14716789 10.1002/bjs.4459

[3] Kawarada O, Sonomura T, Yokoi Y Direct aspiration using rapid-exchange and low-profile device for acute thrombo-embolic occlusion of the superior mesenteric artery. Catheter Cardiovasc Interv 2006; 68: 862–6. 17086541 10.1002/ccd.20827

[4] John S, Hussain MS, Toth G, Bain M, Uchino K, Hui FK Initial experience using the 5MAX™ ACE reperfusion catheter in intra-arterial therapy for acute ischemic stroke. J Cerebrovasc Endovasc Neurosurg 2014; 16: 350–7. 25599043 10.7461/jcen.2014.16.4.350 PMC4296047

[5] McGarry JG, McEvoy SH, Brophy DP Endovascular recanalisation of an acute superior mesenteric artery occlusion. A case report and review of the literature. Ann Med Surg 2015; 4: 76–9. 10.1016/j.amsu.2014.07.005 PMC4372641 25834731

[6] Choi KS, Kim JD, Kim HC, Min SI, Min SK, Jae HJ, *et al* Percutaneous aspiration embolectomy using guiding catheter for the superior mesenteric artery embolism. Korean J Radiol 2015; 16: 736–43. 26175572 10.3348/kjr.2015.16.4.736 PMC4499537

[7] Yang HJ, Cho YK, Jo YJ, Jung YY, Choi SA, Lee SH Successful recanalization of acute superior mesenteric artery thrombotic occlusion with primary aspiration thrombectomy. World J Gastroenterol 2010; 16: 4112–14. 20731029 10.3748/wjg.v16.i32.4112 PMC2928469

[8] Tarr R, Hsu D, Kulcsar Z, Bonvin C, Rufenacht D, Alfke K, *et al* The POST trial: initial post-market experience of the Penumbra system: revascularization of large vessel occlusion in acute ischemic stroke in the United States and Europe. J Neurointerv Surg 2010; 2: 341–4. 21990642 10.1136/jnis.2010.002600

[9] Turk AS, Spiotta A, Frei D, Mocco J, Baxter B, Fiorella D, *et al* Initial clinical experience with the ADAPT technique: a direct aspiration first pass technique for stroke thrombectomy. J Neurointerv Surg 2014; 6: 231–7. 23624315 10.1136/neurintsurg-2013-010713

[10] Shah SN, Sacks D, Chavali R Mechanical embolectomy and recanalization of superior mesenteric artery embolism using the MERCI retrieval device. J Vasc Interv Radiol 2011; 22: 1638–40. 22024124 10.1016/j.jvir.2011.08.007

[11] Tarr R, Hsu D, Kulcsar Z, Bonvin C, Rufenacht D, Alfke K, *et al* The POST trial: initial post-market experience of the Penumbra system: revascularization of large vessel occlusion in acute ischemic stroke in the United States and Europe. J Neurointerv Surg 2011; 3: 97. 10.1136/jnis.2010.002600 21990642

[12] Smith WS, Sung G, Saver J, Budzik R, Duckwiler G, Liebeskind DS, *et al* Mechanical thrombectomy for acute ischemic stroke: final results of the Multi MERCI trial. Stroke 2008; 39: 1205–12. 18309168 10.1161/STROKEAHA.107.497115

